# Large-scale mechanism hypothesis and research prospects of cognitive impairment in schizophrenia based on magnetic resonance imaging

**DOI:** 10.1016/j.heliyon.2024.e25915

**Published:** 2024-02-10

**Authors:** Yue-Wen Gu, Jing-Wen Fan, Shu-Wan Zhao, Xiao-Fan Liu, Hong Yin, Long-Biao Cui

**Affiliations:** a Shaanxi Provincial Key Laboratory of Clinic Genetics, Fourth Military Medical University, Xi’an, China; b Schizophrenia Imaging Lab, Xijing Hospital, Fourth Military Medical University, Xi’an, China; c Department of Radiology, The General Hospital of Western Theater Command, Chengdu, China; d Department of Radiology, Xi'an People's Hospital (Xi'an Fourth Hospital), Xi'an, China; e Department of Radiology, The First Affiliated Hospital of Xi’an Jiaotong University, Xi’an, China

**Keywords:** Schizophrenia, Cognitive impairments, Connectome, Magnetic resonance imaging, Treatment

## Abstract

Cognitive impairments in schizophrenia are pivotal clinical issues that need to be solved urgently. However, the mechanism remains unknown. It has been suggested that cognitive impairments in schizophrenia are associated with connectome damage, and are especially relevant to the disrupted hub nodes in the frontal and parietal lobes. Activating the dorsolateral prefrontal cortex (DLPFC) via repetitive transcranial magnetic stimulation (rTMS) could result in improved cognition. Based on several previous magnetic resonance imaging (MRI) studies on schizophrenia, we found that the first-episode patients showed connectome damage, as well as abnormal activation and connectivity of the DLPFC and inferior parietal lobule (IPL). Accordingly, we proposed that DLPFC-IPL pathway destruction might mediate connectome damage of cognitive impairments in schizophrenia. In the meantime, with the help of multimodal MRI and noninvasive neuromodulation tool, we may not only validate the hypothesis, but also find IPL as the potential intervention target for cognitive impairments in schizophrenia.

Schizophrenia is a chronic mental disorder of unknown etiology with high recurrence and disability, and the Global Burden of Disease released in January 2022 shows it ranks among the top three mental disorders [1]; the China Mental Health Survey shows that the weighted lifetime prevalence of schizophrenia is as high as 0.6% [2]. As the main symptom and core feature, cognitive impairment throughout the course of the illness is a key clinical issue and treatment difficulty [3,4]. Because the pathogenesis has not been fully elucidated [[4], [5], [6]], there are no treatments with significant efficacy for cognitive impairment.

An initial comprehensive search was performed in PubMed. There was no restriction on the publications' date; the search was conducted in September 2023. Since the information was extracted from only one database, the step of removing duplicates was not taken in this study. The keywords were: (((schizophrenia) AND (cognitive impairments or cognitive deficits or cognitive decline or cognitive dysfunction)) AND (connectome or DLPFC or IPL)) AND (MRI). By searching, 234 studies were found, of which 48 articles were included in the present review (Fig. 1).

**Fig. 1:**
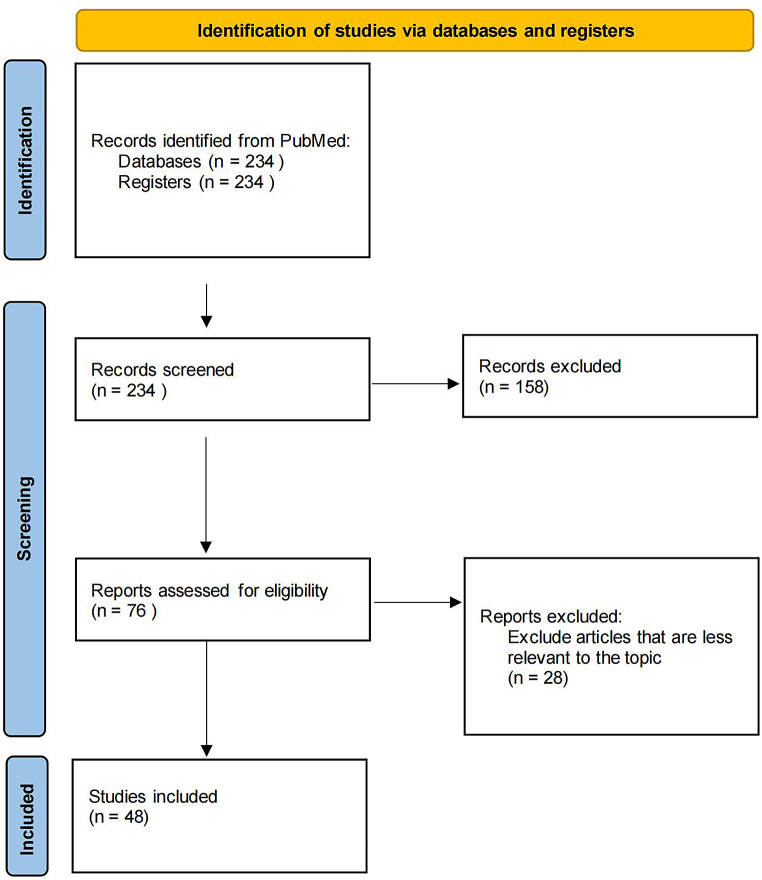
Flowchart: study selection process.

## 1 Cognitive impairment in schizophrenia is a critical clinical difficulty that needs to be addressed

Cognitive impairment is found in approximately 80% of patients with schizophrenia and has a greater impact on disease prognosis and treatment costs than any other symptoms [3]. For more than 100 years, the concept of schizophrenia has evolved from “dementia praecox" to the latest definition in the International Classification of Diseases 11th Revision (ICD-11), but the understanding of the cognitive impairment of the disease has remained constant. *New England Journal of Medicine* and *Lancet* published nine papers entitled “Schizophrenia” one after another in the last 30 years (1994–2022), and it is easy to see that cognitive impairment is gaining more and more attention. Professor René S. Kahn also states that “Schizophrenia Is a Cognitive Illness”, and the origin of schizophrenia is in cognition [6]. Cognitive impairment is now recognized as a central feature of the disease and a major factor in poor prognosis [3].

A meta-analysis of first-episode schizophrenia in China found that patients had deficits in all cognitive domains, including speed of processing, working memory, visual learning, attention/vigilance, verbal learning, reasoning and problem-solving, and social cognition [7]. Also, this is especially true in patients with stable and chronic conditions [8]. Furthermore, longitudinal studies provide ample evidence for the trajectory of cognitive impairment in schizophrenia. A 10-year prospective study showed a progressive decline in cognitive function after the first episode [9]; subsequently, another 20-year cohort study also showed a progressive cognitive decline in patients with psychotic disorders, including schizophrenia [10]. Thus, cognitive impairment is a clinical problem throughout the entire course of schizophrenia.

Over the past decade, the clinical guidelines on schizophrenia have been updated, but have failed to provide clear and consistent guidance on treatment options for cognitive impairment. In terms of new drug development, although some achievements have emerged, there are still many difficulties. According to the Clinical Handbook of Psychotropic Drugs, 23rd edition (Hogrefe, 2019), antipsychotics such as antagonism of D_3_ receptor or 5-hydroxytryptamine receptors may have specific efficacy in improving cognition in the early stages of the disease, but there is no high-level evidence to support this. To improve cognitive symptoms, drugs that enhance cholinergic transmission have received much attention in the 12th edition of The Maudsley Prescribing Guidelines in Psychiatry, but the drug was removed in the next two editions and is as yet unused in clinical practice. The new drug BI 425809 (selective glycine transporter-1 inhibitor) has shown preliminary evidence of improving the cognitive function in schizophrenia and received breakthrough therapy designation by the by the China National Medical Products Administrationin in 2021, but the clinical use of the drug is still dependent on the results of a global multicenter phase III clinical trial [11]. It was found that neuroanatomic heterogeneity exists in antipsychotic-treated patients, with disparate associations with cognition [12].

In summary, cognitive impairment in patients with schizophrenia is a key clinical problem that needs to be addressed, but targeted pharmacotherapy still has shortcomings [5], and existing treatment options are unable to improve patients' cognitive function directly essentially [4]. The unclear pathogenesis remains the crux of the current clinical dilemma.

## 2 Large-scale mechanistic hypothesis of cognitive impairment in schizophrenia

### 2.1 Large-scale mechanisms can help find potential intervention targets and predict treatment efficacy

**Fig. 2:**
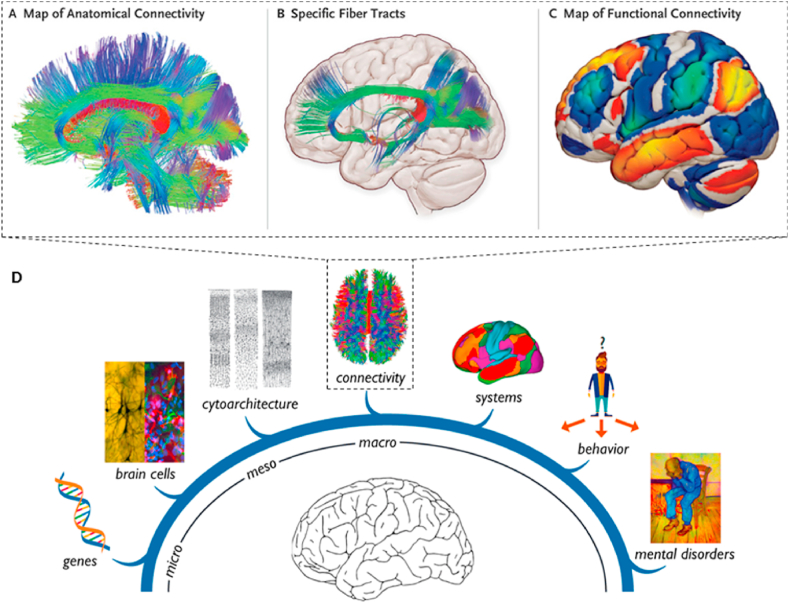
Brain connectome and multiscale neuroscience of mental disorders. (A–C) Anatomical (structural) connections are used to separate fiber tracts and functional connections are used to identify brain areas where spontaneous activity is relevant (adapted from reference [14] with permission). (D) MRI-based, connectomics plays an important role in resolving macro-scale mechanisms of mental disorders (adapted from reference [15] with permission).

In the face of these clinical dilemmas, exploration of the pathogenesis of cognitive impairment in schizophrenia has been ongoing. Large-scale network research is one of the most potentially impactful emerging areas [13]. The human brain is composed of approximately 100 billion neurons, which form complex connections with each other and constitute a unique network for information processing in the nervous system; large-scale networks, on the other hand, are mainly composed of structural and functional connections based on MRI, and connectomics allows for more accurate brain mapping with higher resolution, making the study of large-scale networks a powerful approach to understanding the disease (Fig. 2). The current view is that excitatory pyramidal cells and inhibitory interneurons in the cerebral cortex mediate γ-oscillation abnormalities and network dysfunction, ultimately causing cognitive deficits and negative symptoms in schizophrenia, a theory that also highlights the significance of large-scale network research [4].

In clinical treatment, noninvasive brain stimulation (NIBS) including transcranial magnetic stimulation (TMS) and transcranial direct current stimulation (tDCS) may have procognitive effects, with high tolerability [16] [17]. It was indicated that tDCS was effective and safe in ameliorating negative symptoms in patients with schizophrenia [18]. However tDCS does not have a corresponding recommendation for the treatment of cognitive impairment in schizophrenia currently [19]. Repetitive transcranial magnetic stimulation (rTMS) is a commonly used noninvasive neuromodulation tool for the treatment of psychiatric disorders. rTMS in combination with antipsychotics is one of the main current treatment options for schizophrenia, for hallucinations and negative symptoms (level C recommendation) [20]. A systematic review noted that rTMS with high-frequency stimulation of the left DLPFC is a potentiating technique initially considered to improve cognitive function, especially working memory [21]. Therefore, considering the therapeutic value of noninvasive neuromodulation [[22], [23], [24]], the exploration of MRI-based large-scale mechanisms can also facilitate the discovery of potential targets for stimulation interventions and the development of efficacy prediction models.

### 2.2 Cognitive impairment in schizophrenia is associated with brain connectome disruption

Taking large-scale mechanism analysis as a breaking point, a large number of brain functional and structural connectivity studies based on MRI were carried out earlier [[25], [26], [27], [28], [29], [30]], which fully portrayed the pattern of brain connectome disruption in schizophrenia. Schizophrenia patients have brain connectome disruption, which is associated with impairment of higher cognitive networks such as the frontoparietal network and the default mode network.

**Fig. 3:**
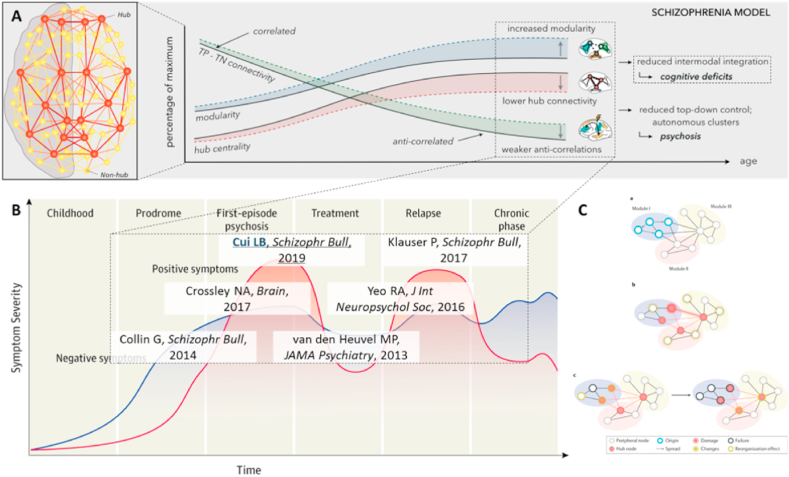
Schizophrenia and brain connectome disruption. (A) The “connectomic model" suggests that reduced integration between modalities during brain connectome development causes cognitive deficits (adapted from reference [33] with permission). (B) Chain of evidence for brain connectome disruption throughout the patient's illness (adapted from reference [4] with permission). (C) Brain connectome disruption has a cascade effect (adapted from reference [34] with permission).

A “connectomic model” has been proposed to address the symptoms of the disease. Connectome studies showed a weakened negative correlation between the default mode network and the frontoparietal network, reduced rich club connectivity, and increased modularity, resulting in cognitive impairment and psychotic symptoms (Fig. 3A). A meta-analysis suggests that the salience processing network (ventral attention network, VAN) plays a central role in the difficulty of patients to differentiate self-representation (inner world) and environmental salience processing (outside world). In addition, hypo-connectivity between the VAN and the frontoparietal network has been found in patients with schizophrenia [31]. Another meta-analysis suggests that the core network might act as a dysfunctional hub of regulation [32]. However, to date, there is no definitive scientific hypothesis for cognitive impairment in schizophrenia, and the following analysis may reveal macro-scale mechanisms of cognitive impairment.

First, a series of MRI connectomic studies have found that schizophrenia exhibits a disruption of the brain connectome throughout the duration of the disease (Fig. 3B) [35]. As a high-risk group, healthy offspring of schizophrenia patients had 7.9% lower rich club connection strength than healthy controls, while patients had 19.6% lower rich club connection than healthy controls, and the longer the duration of untreated psychosis, the lower the rich club connection. The brain structural network rich club organization was damaged in first-episode drug-naïve patients, first-episode treated patients, and chronic patients. And the amount of change in global efficiency of the structural network was positively correlated with drug dose after 12 weeks of antipsychotic treatment in first-episode patients. Meanwhile, connectomic features also correlate with cognitive function; patients with longer characteristic path length of structural network have worse cognitive ability [36]. Moreover, baseline brain connectome-rich club and local connections can predict overall cognitive ability at follow-up, and topological feature clustering coefficients can predict mental retardation [36,37]. The above evidence suggests that cognitive impairment in schizophrenia is closely related to its whole-course brain connectome disruption.

Second, brain connectome disruption has a cascade effect, usually starting with a specific module triggering the involvement of neighboring hub nodes and even more nodes (Fig. 3C) [34]. Structural hub nodes in the brain connectome include precuneus, anterior/posterior cingulate cortex, insula cortex, superior frontal cortex, temporal cortex, and lateral parietal cortex, and functional hub nodes include ventral/dorsal precuneus, anterior/posterior cingulate cortex, ventral medial frontal cortex, and inferior parietal cortex [38]. So, which nodes trigger the disruption of the brain connectome in cognitive impairment in schizophrenia?

### 2.3 Impaired DLPFC-IPL pathway may mediate brain connectome disruption and be involved in cognitive impairment in schizophrenia

The MATRICS Consensus Cognitive Battery (MCCB) is the standardized test for providing a reliable and valid assessment of cognition recommended by U.S. Food and Drug Administration (FDA). The MCCB includes 10 neuropsychological tests clustered in 7 cognitive domains [39], including speed of processing, attention/vigilance, working memory, verbal learning, visual learning, reasoning/problem solving, and social cognition. Cognitive composite scores were used to identify subjects with and without cognitive dysfunction. Our comprehensive review of MRI studies of schizophrenia using MCCB as an assessment tool found that the pivotal nodes of the frontal and parietal lobes were significantly altered in cognitive impairment. Functional MRI studies suggest that patients with declining scores in all seven cognitive domains have reduced frontoparietal network connectivity [40], and patients with higher amplitude of low-frequency fluctuations (ALFF) and lower gray matter volume in DLPFC and higher ALFF in IPL have worse performance in verbal learning and visual learning [41]. The magnetization transfer ratio (MTR) study suggested a positive correlation between prefrontal white matter MTR and MCCB composite score and Stroop score in healthy controls, but this correlation was not seen in patients [42]. Multimodal studies suggested that structural and functional abnormalities in gray and white matter associated with cognitive impairment measured by MCCB composite score are mainly located in DLPFC and IPL [43].

**Fig. 4:**
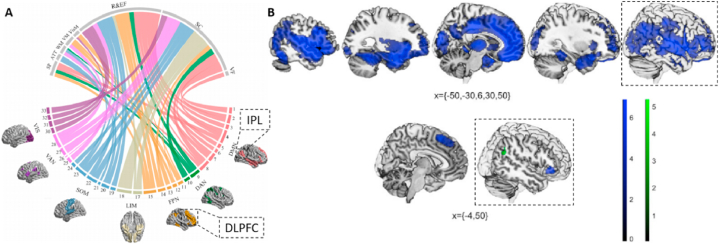
Meta-analyses of cognitive function and brain imaging studies in patients with schizophrenia. (A) gray matter structure in IPL (brain region 4), and DLPFC (brain region 13) is significantly associated with cognitive function (adapted from reference [47] with permission); (B) cognitive tasks are associated with reduced gray matter volume in IPL and DLPFC, as well as reduced functional activity in IPL (adapted from reference [48] with permission).

Once again, impaired DLPFC-IPL pathways in schizophrenia have been confirmed by whole-brain analysis. It was shown that there were differences in a large number of functional connections between first-episode patients and healthy controls, in which the DLPFC-IPL connection was detected [28]; in addition, the abnormal brain regions in the frontoparietal network of patients mainly included the DLPFC and IPL [26]. Besides, in a binary logistic regression model, the right IPL regional homogeneity (ReHo) was one of the significant predictors of auditory verbal hallucinations in schizophrenia [44]. By dynamic causal modeling of effective connectivity, patients had diminished effective connections from the IPL to the DLPFC in working memory tasks [45] while people with high delusional ideation showed a significant increase in DLPFC activation during the working-memory task [46]. According to two recent meta-analyses, IPL and DLPFC were significantly associated with cognitive function in patients (Fig. 4A) [47], and reduced gray matter volume in IPL and DLPFC and reduced functional activity in IPL were associated with cognitive tasks (Fig. 4B) [48]. Notably, increasing the excitability of the DLPFC enhances the cognitive function of schizophrenia patients [21], especially the efficacy of stimulation is significant when combined with neuro-navigation to precisely localize the DLPFC [22].

Therefore, we propose the following hypothesis: impairment of the DLPFC-IPL pathway may mediate the disruption of the brain connectome that is involved in the cognitive impairment of schizophrenia. Based on the hypothesis of the DLPFC-IPL pathway and its effect on the brain connectome, direct intervention of this pathway can be performed in the future with the help of neuro-navigated rTMS combined with MRI for precise localization and stimulation of brain regions; meanwhile, the large-scale (macroscopic) mechanisms of cognitive impairment in schizophrenia can be revealed by brain connectomics based on MRI.

## 3 Prospects for research on the large-scale hypothesis of cognitive impairment in schizophrenia

### 3.1 Discovery of potential intervention targets for cognitive impairment in schizophrenia with the help of noninvasive neuromodulation

Based on the hypotheses, If we can regulate the activity of brain regions in DLPFC or IPL and correct the damaged state of the pathway, it will both prove the above hypothesis and identify potential targets for intervention. In a network meta-analysis, excitatory NIBS protocols over the left DLPFC were associated with significantly large improvements in the severity of negative symptoms. The NIBS protocols includes rTMS, theta-burst stimulation, transcranial random noise stimulation, transcutaneous vagus nerve stimulation, and tDCS [49]. It is worth paying special attention that compared with other NIBS protocols, neuronavigated rTMS guided by MRI for precise localization of stimulated brain regions offers us the possibility of identifying the treatment targets. A recent randomized double-blind sham stimulation controlled study found memory improvement in chronic patients after 8 weeks of treatment with high-frequency neuronavigated rTMS by activating the left DLPFC [22]. In addition, inter-hemispheric homotopic connectivities have a reciprocal inhibitory effect, and inhibition of the right IPL by rTMS exerts a relative activation of the left IPL, which can improve patients' social cognition [50].

Few studies have used MRI with rTMS to explore the neural mechanisms of cognitive impairment in schizophrenia, and structural imaging studies have found no significant changes in cortical thickness after patients received 10 sessions of high-frequency rTMS targeting the bilateral DLPFC [51]. In contrast, in other symptom dimensions, stimulation of the midline cerebellar sites using an intermittent theta-burst stimulation pattern, which is used to strengthen the cerebellar network connections to the right DLPFC, improved the severity of negative symptoms [23]; stimulation of the left temporoparietal joint area using a continuous theta-burst wave caused a reduction in the functional connectivity of the left cerebellum and the whole brain and was able to treat hallucinations [24]. Neuronavigated rTMS can provide both direct evidences to deeply resolve the neural mechanisms behind cognitive impairment in schizophrenia and explore the potential of left-sided IPL as a new target.

### 3.2 MRI cross-omics analysis helps predict the efficacy of cognitive impairment in schizophrenia

Radiomics is an effective method for quantitative analysis and prediction using medical imaging big data, which can significantly improve the effective utilization of medical imaging information, the accuracy of early diagnosis of diseases, and the predictability of treatment outcomes, and has demonstrated excellent performance in imaging multiple organs. The workflow includes high-throughput feature extraction and screening, model building and testing, etc. With the continuous revelation of the biological basis of features, radiomics can provide physicians with sound quantitative recommendations for selecting treatment options [52].

Radiomics entails transforming medical images into high-dimensional data, performing high-throughput extraction, and identifying key features that ultimately support decision-making. Although schizophrenia does not have physical “lesions" for feature extraction, considering abnormal connections as features may be a key point. A successful attempt has been made in the previous study of brain markers and prognosis prediction in schizophrenia based on a radiomics strategy [29,30]. Specifically, the predictive model for overall schizophrenia outcome has twelve features involving nine functional connections predominately included inter-hemispheric connectivity and three brain structural features with a high contribution from the cortical features of IPL [29]. MRI cross-omics analysis may become a more effective and clinically translatable research paradigm.

## 4 Research hypothesis and implications

In summary, focusing on the clinical difficulties of cognitive impairment in schizophrenia, we combined the hot spots and frontiers of MRI and neuromodulation and found that the following problems remain in this field of research: first, no clear macro-scale mechanism hypothesis has been developed theoretically; second, previous studies have not assessed complete cognitive function, the possible pathways were not demonstrated by targeted neuromodulation; third, no new potential intervention targets and efficacy prediction models have been applied.

**Fig. 5:**
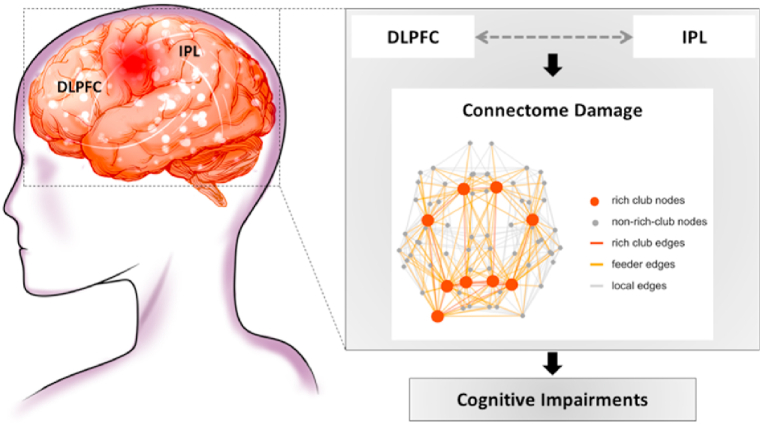
Macro-scale mechanistic hypothesis of cognitive impairment in schizophrenia. Impaired structure and function of the DLPFC-IPL pathway may mediate disruption of the brain connectome and play an important role in cognitive impairment in schizophrenia (adapted from reference [54] with permission).

Therefore, we propose the following hypothesis: impaired DLPFC-IPL pathway mediates brain connectome disruption in cognitive impairment in schizophrenia (Fig. 5). Based on this hypothesis, the large-scale mechanisms of cognitive impairment in schizophrenia can be explored in the future by integrating multimodal MRI connectomic features with the help of neuronavigated rTMS-targeted modulation. Effective connectivity can provide us with the directionality of the brain's functional connectivity [53], which has important implications for the cascade effect of impaired brain connectivity in schizophrenia [34]. The use of multimodal MRI can provide us with more important information about the brain effective connectivity, which in combination with MRI-navigated rTMS can not only validate the hypothesis, but also help us to discover more stimulation targets. An in-depth study of the large-scale mechanisms of cognitive impairment in schizophrenia can provide evidence to support MRI-assisted clinical decision-making, and provide new ideas and directions to solve the current clinical treatment dilemmas of schizophrenia, thus providing important scientific strategies to help schizophrenia patients reduce their disabling conditions and improve their prognosis.

## Ethics approval and consent to participate

None.

## Funding

This work was supported by The National Natural Science Foundation of China, grant number 82271949, 10.13039/501100002858China Postdoctoral Science Foundation, grant number 2020M683739 and the 10.13039/501100007547Key Research and Development Program of Shaanxi Province, grant number 2023-YBSF-444.

## Consent for publication

Yes.

## CRediT authorship contribution statement

**Yue-Wen Gu:** Writing – review & editing, Writing – original draft. **Jing-Wen Fan:** Writing - review & editing. **Shu-Wan Zhao:** Writing - review & editing. **Xiao-Fan Liu:** Writing - Review & editing. **Hong Yin:** Conceptualization, Writing - review & editing. **Long-Biao Cui:** Writing – review & editing, Validation, Conceptualization.

## Declaration of competing interest

The authors declare that they have no known competing financial interests or personal relationships that could have appeared to influence the work reported in this paper.
